# Weight mitigation and tolerability of adjunctive topiramate in acute pediatric psychiatry: a retrospective real-world analysis

**DOI:** 10.3389/fpsyt.2026.1905527

**Published:** 2026-07-22

**Authors:** Binay Kayan Ocakoglu, Aysegul Tonyali, Esra Bulanik Koc, Omca Guney, Seda Demirci, Aslı Ece Soykut, Beyza Mentes, Sukret Alev, Gul Karacetin

**Affiliations:** Department of Child and Adolescent Psychiatry, Bakırköy Prof. Dr. Mazhar Osman Mental Health and Neurological Diseases Training and Research Hospital, Istanbul, Türkiye

**Keywords:** inpatient, pediatric psychiatry, polypharmacy, second-generation antipsychotics, topiramate, weight gain

## Abstract

**Background/objective:**

Psychotropic medications are often associated with significant and rapid weight gain in pediatric populations, especially second-generation antipsychotics and mood stabilizers. This was a retrospective study assessing weight gain, and tolerability of adjunctive topiramate in hospitalized child and adolescent psychiatric patients.

**Methods:**

A total of 285 inpatients were included in the study: an intervention group (n = 45) receiving adjunctive topiramate and a control group (n = 240) receiving standard care. Net weight, polypharmacy burden (chlorpromazine [CPZ] equivalent doses and other mood stabilizers) and psychiatric recovery (Global Assessment of Functioning [GAF] and Clinical Global Impression-Severity [CGI-S] scores) were measured. Hierarchical ANCOVA models were employed to disentangle the independent metabolic effects of topiramate from the confounding effects of polypharmacy.

**Results:**

The mean weight gain in the control group was significantly greater than in the topiramate group (3.3 kg vs -0.1 kg, p < 0.001). This protective metabolic effect persisted with high statistical significance even after controlling for the concurrent CPZ equivalent dose (3.1 kg advantage). Clinical recovery, measured by equivalent improvement in GAF scores and reduction in CGI-S scores, was similar for both groups.

**Conclusions:**

Adjunctive topiramate is a highly effective and safe metabolic shield in acute pediatric inpatient settings. It reduces the risk of psychotropic weight gain and can substantially reduce the need for obesogenic polypharmacy, without impeding the course of psychiatric recovery.

## 1 Introduction

There has been a dramatic increase in the use of psychotropic medications, especially second-generation antipsychotics (SGAs), over the past 20 years to treat children and adolescents with a variety of psychiatric conditions including bipolar disorder, autism spectrum disorder (ASD), and disruptive behavior disorders (1). These pharmacological interventions are very effective for symptom management, but are often associated with serious metabolic adverse effects, especially significant weight gain (2). Emerging data indicate that pediatric patients are particularly susceptible to metabolic changes associated with antipsychotics and are more rapidly gaining weight than adults (3). Such non-age-appropriate weight gain impacts negatively not only on long-term cardiovascular and endocrine morbidities but also on self-esteem, stigmatization and is a major barrier for treatment adherence (4).

The management of the hospitalized pediatric patient is one of the most difficult clinical tasks. Polypharmacy is often required in the acute inpatient setting to achieve stabilization, which in turn multiplies the overall metabolic and side-effect burden (5, 6). In addition, many of these youth, particularly those diagnosed with ASD, show marked impulsivity, agitation and aggressive behaviors (7). An important dilemma in pediatric psychopharmacology is the management of these complex behavioral presentations while attempting to minimize metabolic deterioration, highlighting the need for adjunctive treatments that can simultaneously target both the metabolic and behavioral domains (8).

Topiramate is a broad spectrum anticonvulsant that modulates GABAergic and glutamatergic neurotransmission and has shown promise as an adjunctive agent (9, 10). Numerous studies have demonstrated that topiramate is effective in reducing SGA-associated weight gain and decreasing body mass index (BMI) in children and adolescents (4). In addition to its well known metabolic effects, topiramate has been shown to decrease impulsivity in various psychiatric disorders (11, 12). It has shown promise in the treatment of disruptive and aggressive behaviors in youth with ASD, particularly in pediatric populations (7). In addition, preliminary retrospective data suggest that adjunctive topiramate may improve overall clinical severity and mood symptoms in hospitalized children and adolescents without compromising safety (10).

However, despite these benefits, there is a lack of real-world data evaluating the overall impact of topiramate in acute pediatric settings, particularly its ability to independently reduce metabolic risk and reduce the overall burden of polypharmacy, without compromising psychiatric recovery. The main aim of this retrospective study was therefore to evaluate the metabolic effects and clinical usefulness of adjunctive topiramate in a group of hospitalized child and adolescent psychiatric patients.

## 2 Materials and methods

### 2.1 Study design and populations

This study was a retrospective chart review of child and adolescent patients who were admitted to Prof. Dr. Mazhar Osman Mental Health Training and Research Hospital, Child and Adolescent Psychiatry Clinic, and who received inpatient treatment between 2015 and 2022. This study sample included 285 hospitalized patients. The cohort was subdivided into two groups to assess the clinical and metabolic effects of topiramate: an intervention group of patients who received adjunctive topiramate during their admission (n = 45) and a control group of patients who received standard psychiatric treatment but not topiramate (n = 240). To maximize statistical power for the subsequent covariance analyses, and to reflect accurately the naturalistic prescribing patterns of the inpatient unit, all inpatient admissions during the study period were included, resulting in a naturally larger standard-care control group.

### 2.2 Clinical assessment and data collection

Data were extracted retrospectively from comprehensive patient medical records.

#### 2.2.1 Sociodemographic data

The baseline demographic variables collected were the patient’s age, gender and the educational status.

#### 2.2.2 Clinical and diagnostic data

Primary and comorbid psychiatric diagnoses were identified and categorized into five main groups: Bipolar Disorder, Schizophrenia Spectrum Disorder, Conduct Disorder, Depressive Disorder, and ASD. The duration of illness along with the duration of hospital stay (difference between date of admission and date of discharge) was also noted.

#### 2.2.3 Metabolic outcomes

The main metabolic outcome measure was net weight change, which was calculated by subtracting the patient’s admission weight from their discharge weight.

#### 2.2.4 Psychiatric efficacy and severity

Clinical recovery was assessed using the Global Assessment of Functioning (GAF) and Clinical Global Impression-Severity (CGI-S) scales at admission and discharge, in order to determine that adjunctive treatment did not hinder psychiatric stabilization.

The study protocol was approved by the Bakirkoy Sadi Konuk Training and Research Hospital Ethics Committee (approval number: 2022-04-03). All procedures were conducted in accordance with the latest version of the Declaration of Helsinki. Informed consent was obtained from all patients prior to hospitalization.

### 2.3 Pharmacological variables and polypharmacy adjustments

Detailed psychopharmacological data were recorded at discharge for all patients. To ensure clinical and metabolic relevance of recorded medications, a minimum duration criterion was applied; only medications prescribed consistently for at least two weeks before discharge were included in the analysis.

- All prescribed antipsychotic medications were converted to Chlorpromazine (CPZ) equivalent doses (in mg) to account for the significant metabolic burden of concurrent antipsychotic use using standard literature-based conversion factors.
- Also, for each patient, the total number of concomitant classic mood stabilizers (eg, lithium, valproate, carbamazepine, oxcarbazepine, and lamotrigine) was calculated to quantify the overall polypharmacy burden and to isolate the independent effect of topiramate.
- Specifically, for the topiramate group the final discharge dose of topiramate (mg/day) was recorded to assess possible dose-response relationships.

### 2.4 Statistical analysis

All statistical analyses were performed using the R software environment.

- Descriptive statistics were presented as means, standard deviations, and medians for continuous variables, and as frequencies and percentages for categorical variables.
- Appropriate parametric or non-parametric tests were used for baseline comparison between topiramate and control groups depending on the distribution of data.
- A one-way Analysis of Variance (ANOVA) with Tukey *post-hoc* test was used to test weight change difference between diagnostic categories. Hierarchical analyses of covariance (ANCOVA) were performed to disentangle the independent metabolic effect of topiramate from the confounding effects of polypharmacy. These models were statistically adjusted for the CPZ equivalent dose and the total number of additional mood stabilizers.
- Finally, we conducted a multiple linear regression analysis restricted to the topiramate subgroup to examine the dose-response relation between dose of topiramate and weight change.
- The level of statistical significance was set at p < 0.05 for all analyses.

## 3 Results

### 3.1 Sample characteristics and baseline demographics

The study included a total of 285 hospitalized pediatric and adolescent patients. The mean age of the patients was 15.8 ± 2.0 years. The cohort consisted of 156 (55%) males and 129 (45%) females. Forty-five patients (15.8%) of the total sample were treated with the add-on topiramate and 240 patients (84.2%) were treated with the standard psychiatric treatment (control group). There were no statistically significant differences between the topiramate and control groups regarding age (15.4 ± 1.9 vs. 15.9 ± 2.0 years, *p* = .056) or gender distribution (*p* = .70). However, patients in the topiramate group had a significantly higher baseline (admission) weight compared to the control group (81 ± 22 kg vs. 67 ± 15 kg, *p* <.001). In addition, the topiramate group had a significantly longer duration of illness compared to the control group (70.7 ± 53.6 vs. 30.7 ± 39.4 months, p <.001) (**Table 1**). Among the patients receiving adjunctive topiramate, the mean daily dose at discharge was 136.6 ± 75.3 mg (median: 100 mg, range: 25–300 mg).

**Table 1:** Baseline demographic and clinical characteristics of the study population.

Characteristic	Overall (N = 285)	Control group (N = 240)	Topiramate group (N = 45)	p-value
Age (Years), mean (SD)	15.8 (2.0)	15.9 (2.0)	15.4 (1.9)	.056
Gender, n (%)				
Male	156 (55%)	130 (54%)	26 (58%)	.700
Female	129 (45%)	110 (46%)	19 (42%)	
Educational status, n (%)				
Primary Education	46 (16%)	36 (15%)	10 (23%)	.061
High School	114 (40%)	103 (43%)	11 (25%)	
Open High School	39 (14%)	29 (12%)	10 (23%)	
Out of School	83 (29%)	70 (29%)	13 (30%)	
Weight at Admission (kg), mean (SD)	68.8 (17.2)	66.7 (15.2)	80.5 (22.3)	<.001*
Duration of Illness (Months), mean (SD)	38.2 (45.1)	30.7 (39.4)	70.7 (53.6)	<.001*
Length of Hospital Stay (Days), mean (SD)	42.3 (29.5)	44.7 (30.04)	29.3 (22.9)	<.001*
Topiramate dose, mean (SD)			136.6 ± 75.3 Median: 100 mg Range: 25–300 mg	

SD, Standard Deviation. Categorical variables are presented as n (%), and continuous variables are presented as mean (SD). * indicates statistical significance (p < 0.05).

### 3.2 Diagnostic and pharmacological profiles

Regarding diagnostic distribution, significant differences were observed between the groups (*p* <.001) (**Table 2**). *Post-hoc* analyses (see **Supplementary Table 1**) revealed that the prevalence of Conduct Disorder (*Z* = 2.84, *p* <.05) and Autism Spectrum Disorder (*Z* = 4.10, *p* <.05) was significantly higher in the topiramate group. Conversely, the prevalence of Bipolar Disorder was significantly lower in the topiramate group compared to the control group (*Z* = -5.32, *p* <.05).

**Table 2:** Diagnostic profile of the patient cohort.

Primary diagnosis category	Overall (N = 285)	Control group (N = 240)	Topiramate group (N = 45)	P-value
Bipolar Disorder, n (%)	160 (56.1%)	151 (63%)	9 (20%)	<.001*
Schizophrenia Spectrum, n (%)	26 (9.1%)	20 (8.3%)	6 (13%)	
Conduct Disorder, n (%)	57 (20%)	41 (17%)	16 (36%)	
Autism Spectrum Disorder (ASD), n (%)	15 (5.2%)	7 (2.9%)	8 (18%)	
Depressive Disorder, n (%)	27 (9.4%)	21 (8.8%)	6 (13%)	

* indicates statistical significance (p < 0.05).

CPZ equivalents were significantly lower in the topiramate group (710 ± 514 mg/day) than in the control group (960 ± 642 mg/day) (p = 0.016), representing a significantly lower pharmacological load. Despite this difference in overall dosage, both groups were exposed to comparable rates of antipsychotic polypharmacy (p = 0.300; multiple antipsychotic use: 69% in the control group and 58% in the topiramate group). However, a highly significant difference was observed regarding the use of additional mood stabilizers (p < 0.001). While 98.7% of the control group was prescribed at least one additional mood stabilizer (e.g., lithium, valproate), topiramate was preferred as the sole mood-stabilizing strategy for 47% of the patients in the topiramate group (**Table 3**).

**Table 3:** Pharmacological load and polypharmacy characteristics.

Pharmacological parameter	Overall cohort (N = 285)	Control group (N = 240)	Topiramate group (N = 45)	p-value
Mean CPZ Equivalent Dose (mg/day), mean (SD)	921 (630)	960 (642)	710 (514)	0.016*
Multiple Antipsychotic Use (≥2 agents), %	–	69%	58%	0.300
Additional Mood Stabilizer Use (≥1 agent), %	–	98.7%	53%	<0.001*
Sole Mood-Stabilizing Strategy (Monotherapy equivalent), %	–	1.3%	47%	<0.001*
Specific antipsychotic agents, n (%)				
Risperidone	169 (59%)	151 (63%)	18 (40%)	0.004*
Olanzapine	41 (14%)	37 (15%)	4 (8.9%)	0.3
Quetiapine	179 (63%)	156 (65%)	23 (51%)	0.077
Aripiprazole	41 (14%)	35 (15%)	6 (13%)	0.8
Amisulpride	32 (11%)	19 (7.9%)	13 (29%)	<0.001*
Haloperidol	12 (4.2%)	8 (3.3%)	4 (8.9%)	0.10
Specific classic mood stabilizers, n (%)				
Valproate	143 (50%)	128 (53%)	15 (33%)	0.014*
Lithium	130 (46%)	122 (51%)	8 (18%)	<0.001*
Carbamazepine	3 (1.1%)	2 (0.8%)	1 (2.2%)	0.4
Oxcarbazepine	1 (0.4%)	0 (0%)	1 (2.2%)	0.2
Lamotrigine	17 (6.0%)	17 (7.1%)	0 (0%)	0.084

CPZ, Chlorpromazine. SD, Standard Deviation. Data are presented as mean (SD) for continuous variables and percentages for categorical variables. * indicates statistical significance (p < 0.05).

**Table 3** describes the distribution of individual psychotropic medications in both cohorts. There was a significant difference in the use of specific classic mood stabilizers between the groups. The control group used valproate significantly more often than the topiramate group (53% vs 33%, p=0.014) and lithium (51% vs 18%, p<0.001). For second generation antipsychotics (SGAs) there was a significantly higher use of risperidone in the control group (63% vs 40%, p=0.004). Use of quetiapine (65% vs. 51%, p=0.077) and olanzapine (15% vs. 8.9%, p=0.30) were also higher in the control group but the differences were not statistically significant. The use of amisulpride was significantly more common in the topiramate group (29% vs 7.9%, p<0.001). There were no significant differences between groups in distribution of aripiprazole (15% vs 13%, p=0.80) and haloperidol (3.3% vs 8.9%, p=0.10).

By comparing the admission and discharge weights of the groups it was found that the baseline (admission) weight was significantly higher in the topiramate group (81 ± 22 kg) than in the control group (67 ± 15 kg) (p < 0.001). Net weight change (weight on discharge minus weight on admission) was different during the treatment period (p < 0.001). During this period, the patients of the control group, receiving standard treatment, gained an average of 3.3 ± 5.2 kg. In contrast, patients given topiramate and their treatment regimens did not gain weight (-0.1 ± 4.6 kg) and stayed at their baseline weights (**Table 4**).

**Table 4:** Comparison of weight measurements between the topiramate and control groups.

Measurement	Overall cohort (N = 285)	Control group (N = 240)	Topiramate group (N = 45)	p-value
Admission Weight (kg)	69 ± 17 (Median: 67, Range: 35 - 133)	67 ± 15(Median: 65, Range: 35 - 114)	81 ± 22 (Median: 78, Range: 42 - 133)	<0.001*
Discharge Weight (kg)	71 ± 16 (Median: 70, Range: 35 - 129)	69 ± 15 (Median: 69, Range: 35 - 113)	78 ± 20 (Median: 76, Range: 43 - 129)	0.026*
Weight Change (kg) [Discharge - Admission]	2.8 ± 5.2 (Median: 2.0, Range: -18.0 to 26.0)	3.3 ± 5.2 (Median: 3.0, Range: -18.0 to 26.0)	-0.1 ± 4.6(Median: 0.0, Range: -10.0 to 11.0)	<0.001*

Data are presented as mean ± standard deviation.). * indicates statistical significance. (p <0.05).

### 3.3 Independent metabolic effects of topiramate: adjusting for antipsychotic and mood stabilizer burden

ANCOVA were conducted to evaluate the independent effect of topiramate on weight change, controlling for the metabolic burden of polypharmacy.

The only covariate included in Model 1 was the dose of CPZ equivalent. The model revealed a statistically significant main effect of CPZ dose on weight gain. Importantly, this antipsychotic burden was statistically controlled for and yet topiramate’s protective effect on weight change remained highly significant (average weight advantage of 3.1 kg compared with controls).

In Model 2, the total number of additional mood stabilizers (e.g., lithium, valproate) was added as a second covariate to account for the full pharmacological load. In this model, the number of concurrent mood stabilizers emerged as a profound and independent predictor of weight gain (adding an average of 1.4 kg per additional agent). The inclusion of mood stabilizers overshadowed the effect of the CPZ equivalent dose, which consequently lost its statistical significance. Even under these conditions, topiramate maintained a borderline significant protective trend, affording a 2.0 kg metabolic advantage despite the heavy polypharmacy burden (**Table 5**).

**Table 5:** Hierarchical ANCOVA models evaluating predictors of weight change.

Predictor	Model 1 estimate (β)	Model 1 p-value	Model 2 estimate (β)	Model 2 p-value
Topiramate Use (Reference: Control)	-3.10	0.002*	-2.02	0.063
CPZ Equivalent Dose (per 1 mg)	1	0.028*	1	0.159
Additional Mood Stabilizers (per 1 agent)	–	–	1.40	0.020*

The dependent variable for both models is net weight change (kg). Model 1 adjusts solely for the chlorpromazine (CPZ) equivalent dose of antipsychotics. Model 2 hierarchically introduces the total number of additional mood stabilizers (e.g., lithium, valproate) to account for the full polypharmacy burden. β represents the unstandardized regression coefficient, indicating the mean weight change in kilograms associated with the predictor.

* indicates statistical significance.

### 3.4 Weight change according to diagnostic groups

To determine whether the effect of topiramate on weight change varied depending on the patient’s underlying psychiatric diagnosis, ANOVA was conducted. The overall model revealed a statistically significant difference in weight change among the five diagnostic groups (F (4, 27) = 3.64, p = 0.0169) (see **Supplementary Table 2**). Descriptive statistics showed that patients diagnosed with Bipolar Disorder experienced an average weight gain of 5.1 kg. In contrast, patients with Conduct Disorder experienced an average weight loss of 2.1 kg while using the medication (see **Supplementary Table 3**).

To identify which specific groups differed from one another, a Tukey *Post-Hoc* test was performed. The *post-hoc* analysis demonstrated that the weight change in patients with Conduct Disorder was significantly different from that of patients with Bipolar Disorder (p = 0.008). (see **Supplementary Table 4**).

### 3.5 Weight change by diagnostic groups and the masking effect of polypharmacy

To determine whether the previously observed differences in weight change among diagnostic groups were confounded by polypharmacy, an Analysis of Covariance (ANCOVA) was conducted. The chlorpromazine (CPZ) equivalent dose of antipsychotics and the total number of additional mood stabilizers were entered into the model as covariates.

The ANCOVA model revealed that both the CPZ equivalent dose [F (1, 25) = 7.17, p = 0.013] and the number of concurrent mood stabilizers [F (1, 25) = 4.37, p = 0.047] exerted significant independent effects on weight change (**Table 6**). Importantly, after controlling for these pharmacologic covariates statistically, the main effect of diagnostic group on weight change was no longer significant statistically [F (4, 25) = 2.62, p = .059]. **Supplementary Table 5** gives detailed parameter estimates including coefficients and standard errors for each diagnostic category.

**Table 6:** Analysis of covariance (ANCOVA) for weight change controlling for pharmacologicl load.

Source of variation	df	Sum of squares	Mean square	F-statistic	p-value
CPZ Equivalent Dose	1	98.22	98.22	7.17	0.013*
Additional Mood Stabilizers	1	59.88	59.88	4.37	0.047*
Diagnostic Group	4	143.76	35.94	2.62	0.059
Residuals	25	342.51	13.70		

CPZ, Chlorpromazine. * indicates statistical significance (p < 0.05).

### 3.6 Dose-response relationship in topiramate users

In a dose-response multiple regression analysis conducted exclusively within the topiramate subgroup (n = 45), a negative trend was observed between the topiramate dose and weight change (= -0.014), indicating increased weight loss with higher doses (**Table 7**). However, this relationship did not reach statistical significance (p = 0.191). Conversely, even within this specific subgroup, the concurrent antipsychotic burden [chlorpromazine (CPZ) equivalent dose] remained a highly significant and independent predictor of weight gain (p = 0.002).

**Table 7:** Multiple regression analysis evaluating the dose-response relationship in topiramate users.

Predictor	Estimate (β)	Standard error	T-statistic	P-value
Intercept	-1.77	1.58	-1.12	0.272
Topiramate Dose (mg)	-0.014	0.11	-1.34	0.191
CPZ Equivalent Dose (mg)	0.006	0.002	3.52	0.002*
Additional Mood Stabilizers	0.238	1.189	0.20	0.843

The dependent variable is net weight change (kg). CPZ, Chlorpromazine. * indicates statistical significance (p < 0.05).

### 3.7 Clinical efficacy and reliability

The clinical severity and functional levels of the groups at psychiatric admission were found to be comparable (Admission GAF p = 0.400, Admission CGI-S p = 0.700). When evaluating the extent of clinical improvement at discharge, the increase in GAF functioning scores (Control: 27 ± 11 vs. Topiramate: 25 ± 13, p = 0.300) and the reduction in CGI severity scores occurred at statistically similar rates in both groups (see **Supplementary Table 5**).

## 4 Discussion

The primary objective of this retrospective study was to evaluate the clinical utility, metabolic outcomes, and tolerability of adjunctive topiramate in a cohort of hospitalized child and adolescent psychiatric patients. Our analyses yielded several findings relevant to current pediatric psychopharmacology. First, adjunctive topiramate was associated with a mitigating effect on the weight gain frequently observed during inpatient psychiatric treatment. Second, this metabolic profile remained statistically significant after adjusting for the concurrent antipsychotic burden, suggesting a potential independent pharmacological benefit. Third, topiramate use was associated with a relatively lower polypharmacy load; nearly half of the patients receiving topiramate were stabilized without the need for additional classic mood stabilizers. Fourth, observational data suggested the clinical preference of topiramate in patients with Conduct Disorder and ASD, which may be attributed to its usefulness in managing impulsivity with high baseline metabolic risks. Importantly, these metabolic effects did not appear to interfere with psychiatric recovery, with similar functional improvements and reductions in symptoms as seen in the standard care group. Collectively, these results suggest that adjunctive topiramate may be a useful and tolerable option for the management of metabolic risks in pediatric inpatient settings.

An aspect of our results that is relevant is in the baseline clinical characteristics and in the dynamics of hospitalization of the sample. While the treatment groups were demographically similar in age and gender, patients prescribed adjunctive topiramate presented with a significantly longer duration of illness and higher admission weights. The significantly higher baseline weight and longer illness duration in the topiramate group reflect the nature of retrospective real-world data. As noted by Martínez-Ortega et al. (6), the physician’s treatment decision in clinical practice is frequently based on the patient’s current weight (confounding by indication). Patients with a longer duration of illness probably experienced more weight gain secondary to long-term psychotropic exposure and polypharmacy (6), which led clinicians to choose adjunctive treatments that improve the metabolic profile such as topiramate (9, 13).

In terms of the diagnostic distribution, there was a significantly higher prevalence of Conduct Disorder and ASD in the topiramate group. This clinical trend can be explained by the increased susceptibility of children with ASD and conduct disorders to weight gain through SGAs (6), and the clinicians’ willingness to capitalize on the potential secondary benefits of topiramate on aggression and impulsivity (7, 14). However, the risk of paradoxical hyperactivity and agitation related to the use of topiramate in patients with ASD should not be underestimated (7). On the other hand, the lower incidence of Bipolar Disorder in the topiramate group is consistent with the literature. Topiramate is often studied for its metabolic benefits (Arman and Haghshenas, 2022), but does not show additional efficacy in controlling symptoms during acute manic episodes (6). Therefore, clinicians tend to prioritize standard, evidence-based mood stabilizers in this patient population.

The topiramate group also presented with a more severe baseline profile in terms of chronicity and weight, their mean length of stay was significantly shorter compared to the standard care group (29.3 vs. 44.7 days). However, this finding must be interpreted with caution. The standard care group also had a significantly higher prevalence of Bipolar Disorder, which by nature requires longer inpatient stabilization periods for acute manic episodes. By contrast, the topiramate group was mostly composed of patients with Conduct Disorder and ASD. Therefore, the reduced length of hospital stay in the topiramate group likely reflects these baseline diagnostic differences rather than an accelerated stabilizing effect of the drug per se.

The overall pharmacological load showed a similar confounding effect in diagnosis. The control group was largely made up of patients with Bipolar Disorder, in which acute manic episodes are designed to require aggressive interventions. The patients received much higher chlorpromazine (CPZ) equivalent doses and concomitant classic mood stabilizers. This combined metabolic risk was reflected in their significantly higher use of obesogenic agents, particularly valproate (53%) and lithium (51%), and in a high prevalence of quetiapine (65%). The topiramate group was on the other hand mainly composed of patients with Conduct Disorder and ASD, where alternative mood-stabilizing strategies and different antipsychotic profiles, such as significantly higher amisulpride use (29%), are usually sufficient for behavioral control. Importantly, although the distribution of weight-gaining medications at baseline was highly uneven across the cohorts, our hierarchical ANCOVA models successfully isolated topiramate’s effect by statistically controlling for both CPZ equivalent doses and the number of concurrent mood stabilizers. Thus, our results confirm an independent ‘metabolic shield’ effect of topiramate in a real-world pediatric setting, protecting against weight gain despite significantly different baseline prescribing patterns.

In our study, the control group with standard treatment gained an average of 3.3 kg, whereas the topiramate-augmented group prevented weight gain, maintaining their baseline weight (-0.1 kg). This finding is consistent with the conclusion of Arman et al. (4), that topiramate stabilizes the patient by preventing antipsychotic-induced weight gain, rather than causing an absolute weight loss. Our ANCOVA model correcting for the chlorpromazine (CPZ) equivalent dose (Model 1) shows that topiramate has a protective effect of 3.1 kg that is strikingly similar to the 2.80 kg mean difference obtained in the recent meta-analysis (1).

The large disparity in the number of concurrent mood stabilizers between the cohorts should be carefully considered. The use of more psychotropic agents naturally encourages more weight gain. Addition of further mood stabilizers like lithium and valproate in our hierarchical ANCOVA model (Model 2) showed an even more pronounced and independent impact on weight gain (+1.4kg per agent) than CPZ dose alone when looking at this. Martínez-Ortega et al. (6) indicate that the concomitant use of antipsychotics with other psychotropics like valproate dramatically increases the risk of obesity in children and adolescents. In the literature, this scenario created by the concurrent use of antipsychotics and mood stabilizers is described as a “double hit”. Topiramate has the potential to attenuate the excessive weight gain caused by combinations such as lithium and risperidone (15). In our study, the observation that topiramate exhibited a statistically significant, but borderline, metabolic advantage of 2.0 kg even after statistically accounting for severe polypharmacy burden indicates that the weight mitigation seen is not just an artifact of the experimental group taking fewer medications, but a real protective barrier. Moreover, the capacity of topiramate to act as a sole mood-stabilizing modality for nearly half of its users (47%) emphasizes a significant indirect clinical benefit: by significantly reducing the overall burden of polypharmacy, it effectively protects vulnerable youth from the cumulative obesogenic effects of such combined treatments.

In our study, the effect of topiramate on weight change initially appeared to vary significantly depending on the underlying psychiatric diagnosis; patients with Conduct Disorder (CD) lost an average of 2.1 kg, whereas patients with Bipolar Disorder (BD) gained an average of 5.1 kg. However, our ANCOVA model revealed that this difference was driven by diagnosis-specific pharmacological burden rather than a biological response. When the chlorpromazine (CPZ) equivalent dose and the number of additional mood stabilizers were statistically controlled, the main effect of diagnosis on weight change lost its significance (p=0.059). This finding is highly consistent with the existing literature. In youth, antipsychotic polypharmacotherapy, especially antipsychotics in combination with mood stabilizers (e.g., lithium, valproate that are often used in BD), has a significantly higher risk of weight gain than monotherapy (6). We found that topiramate can successfully induce weight loss (-2.1 kg) in the absence of heavy mood stabilizer polypharmacy such as Conduct Disorder and this is consistent with the findings of Shapiro et al. (14). Conversely, the heavy polypharmacy burden inherent to Bipolar Disorder treatment masks and limits this protective effect of topiramate.

### 4.1 Limitations of the study

Several limitations must be considered when interpreting the findings of this study. First, the retrospective, observational design inherently precludes the establishment of definitive causal relationships and introduces the risk of selection bias and confounding by indication. As previously noted, topiramate was not assigned randomly but was systematically preferred for patients with higher baseline weights and more chronic illness profiles. Second, the topiramate cohort was relatively small (n = 45) compared to the standard care group (n = 240), which may have limited the statistical power to detect subtler effects, such as a definitive dose-response relationship. Third, while clinicians likely used topiramate for secondary effects on behavioral dysregulation, we did not include specific scales for impulsivity or aggression in our study. Consequently, the impact of topiramate on behavioral domains could only be inferred indirectly through general severity measures (CGI-S) and diagnostic trends. Finally, our data reflect only the acute inpatient stabilization period. Therefore, the long-term sustainability of topiramate’s metabolic protective effects and its safety profile in outpatient settings remain to be elucidated in future prospective, randomized controlled trials.

## 5 Conclusion

This real-world retrospective study highlights the need of adjunctive topiramate as a highly effective and safe “metabolic shield” in acute inpatient management of pediatric psychiatric patients. Topiramate is effective in prevention of the often marked weight gain associated with standard antipsychotic and mood stabilizer treatment. The metabolic advantage of adjunctive topiramate was maintained regardless of the concomitant antipsychotic burden. Moreover, the addition of topiramate resulted in a significant reduction in the overall polypharmacy load, allowing stabilization of a large number of patients without the need for other obesogenic classic mood stabilizers. Importantly, these pharmacologic and metabolic benefits were achieved without compromising the course of psychiatric recovery as evidenced by similar improvements in global functioning and symptom severity. However, the concomitant antipsychotic dosage should be carefully monitored by clinicians to not compromise this protective effect. Adjunctive topiramate is thus an important and well-tolerated clinical strategy to ameliorate the severe metabolic consequences of pediatric psychopharmacotherapy.

## Data Availability

The raw data supporting the conclusions of this article will be made available by the authors, without undue reservation.
