# Supplementary material for: Weight mitigation and tolerability of adjunctive topiramate in acute pediatric psychiatry: a retrospective real-world analysis
Source: Front Psychiatry. 2026 Jul 22;17:1905527. doi: 10.3389/fpsyt.2026.1905527 (PMC13439229; doi:10.3389/fpsyt.2026.1905527)
Supplement: Supplementary file 1 [file SupplementaryFile1.docx]

Supplementary Table 1. Post-Hoc Standardized Residual (Z-Score) Analysis of Diagnostic Categories

| **Diagnostic Category** | **Z-Score** | **Result (Significance)** |
| --- | --- | --- |
| Bipolar Disorder | -5.32 | Significantly LOWER (*p* < .05) |
| Schizophrenia Spectrum Disorder | 1.07 | No Significant Difference |
| Conduct Disorder | 2.84 | Significantly HIGHER (*p* < .05) |
| Depressive Disorder | 0.96 | No Significant Difference |
| Autism Spectrum Disorder (ASD) | 4.10 | Significantly HIGHER (*p* < .05) |

Note. A Z-score absolute value > 1.96 indicates statistical significance at p < .05. Positive values indicate higher prevalence in the topiramate group, while negative values indicate lower prevalence.

Supplementary Table 2. ANOVA Results for Weight Change Across Diagnostic Groups

| Term | df | Sum of Squares | Mean Square | F-Statistic | p-value |
| --- | --- | --- | --- | --- | --- |
| Diagnostic Group | 4 | 225.83 | 56.46 | 3.64 | **0,016** |
| Residuals | 27 | 418.54 | 15.50 |  |  |

Supplementary Table 3. Weight Change by Diagnostic Group

| **Diagnostic Group** | **N** | **Mean Admission Weight (kg)** | **Mean Discharge Weight (kg)** | **Mean Weight Change (kg)** |
| --- | --- | --- | --- | --- |
| 1. Bipolar Disorder | 6 | 60.7 | 65.8 | 5.1 |
| 2. Schizophrenia Spectrum Disorder | 3 | 85.3 | 86.7 | 1.3 |
| 3. Conduct Disorder | 12 | 82.4 | 80.2 | -2.1 |
| 4. Depressive Disorder | 4 | 90.8 | 89.5 | -1.2 |
| 5. Autism Spectrum Disorder (ASD) | 7 | 75.1 | 74.3 | -0.9 |

Supplementary Table 4. Tukey Post-Hoc Test Results (Pairwise Comparisons)

| Contrast | Estimate | Lower CI | Upper CI | Adjusted p-value |
| --- | --- | --- | --- | --- |
| Schizophrenia Spectrum - Bipolar Disorder | -3.75 | -11.88 | 4.38 | 0,665 |
| Conduct Disorder - Bipolar Disorder | -7.21 | -12.96 | -1.46 | **0,008*** |
| Depressive Disorder - Bipolar Disorder | -6.33 | -13.76 | 01.09 | 0,122 |
| Autism Spectrum Disorder - Bipolar Disorder | -5.94 | -12.34 | 0.46 | 0,078 |
| Conduct Disorder - Schizophrenia Spectrum | -3.46 | -10.88 | 3.96 | 0,656 |
| Depressive Disorder - Schizophrenia Spectrum | -2.58 | -11.37 | 6.20 | 0,909 |
| Autism Spectrum Disorder - Schizophrenia Spectrum | -2.19 | -10.13 | 5.74 | 0,926 |
| Depressive Disorder - Conduct Disorder | 0.88 | -5.76 | 7.51 | 0,995 |
| Autism Spectrum Disorder - Conduct Disorder | 1.27 | -4.20 | 6.74 | 0,959 |
| Autism Spectrum Disorder - Depressive Disorder | 0.39 | -6.81 | 7.60 | 0,999 |

* indicates statistical significance (p < 0.05).

Supplementary Table 5. Parameter Estimates of the ANCOVA Model for Weight

| Parameter | Estimate (β) | Standard Error | t-statistic | p-value |
| --- | --- | --- | --- | --- |
| Intercept | 2.81 | 2.17 | 1.30 | 0.207 |
| CPZ Equivalent Dose | 0.004 | 0.0018 | 2.21 | 0.037* |
| Additional Mood Stabilizers | 0.28 | 1.31 | 0.22 | 0.831 |
| Diagnostic Groups (Reference: Bipolar Disorder) |  |  |  |  |
| Schizophrenia Spectrum Disorder | -5.44 | 3.05 | -1.78 | 0.086 |
| Conduct Disorder | -6.74 | 2.14 | -3.15 | 0.004* |
| Depressive Disorder | -5.90 | 2.54 | -2.33 | 0.028* |
| Autism Spectrum Disorder | -5.16 | 2.14 | -2.41 | 0.024* |

Note: Bipolar Disorder serves as the reference category for diagnostic groups in the regression matrix.

Supplementary Table 6. Comparison of Clinical Efficacy and Functional Outcomes Between the Topiramate and Control Groups

| Clinical Measure | Overall Cohort (N=285) | Control Group (N=240) | Topiramate Group (N=45) | p-value |
| --- | --- | --- | --- | --- |
| Admission GAF Score | 15 ± 7 (Median: 14) | 15 ± 7 (Median: 15) | 15 ± 7 (Median: 13) | 0.400 |
| Discharge GAF Score | 42 ± 11 (Median: 43) | 42 ± 11 (Median: 43) | 39 ± 13 (Median: 41) | 0.400 |
| GAF Score Change | 26 ± 11 (Median: 28) | 27 ± 11 (Median: 28) | 25 ± 13 (Median: 27) | 0.300 |
| Admission CGI-S Score, n (%) |  |  |  | 0.700 |
| 4 (Moderately ill) | 2 (0.7%) | 2 (0.9%) | 0 (0%) |  |
| 5 (Markedly ill) | 28 (10%) | 21 (9.2%) | 7 (16%) |  |
| 6 (Severely ill) | 92 (34%) | 77 (34%) | 15 (33%) |  |
| 7 (Extremely ill) | 150 (55%) | 127 (56%) | 23 (51%) |  |
| 9 (Not assessed) | 1 (0.4%) | 1 (0.4%) | 0 (0%) |  |
| Discharge CGI-S Score, n (%) |  |  |  | 0.068 |
| 2 (Borderline ill) | 9 (3.3%) | 8 (3.5%) | 1 (2.3%) |  |
| 3 (Mildly ill) | 100 (37%) | 90 (40%) | 10 (23%) |  |
| 4 (Moderately ill) | 84 (31%) | 70 (31%) | 14 (32%) |  |
| 5 (Markedly ill) | 51 (19%) | 38 (17%) | 13 (30%) |  |
| 6 (Severely ill) | 20 (7.4%) | 17 (7.5%) | 3 (6.8%) |  |
| 7 (Extremely ill) | 7 (2.6%) | 4 (1.8%) | 3 (6.8%) |  |
| CGI-S Score Change, n (%) |  |  |  | 0.047 |
| -5 (Highest reduction) | 3 (1.1%) | 3 (1.3%) | 0 (0%) |  |
| -4 | 52 (19%) | 48 (21%) | 4 (9.1%) |  |
| -3 | 83 (31%) | 74 (33%) | 9 (20%) |  |
| -2 | 73 (27%) | 56 (25%) | 17 (39%) |  |
| -1 | 51 (19%) | 40 (18%) | 11 (25%) |  |
| 0 (No change) | 9 (3.3%) | 6 (2.6%) | 3 (6.8%) |  |

(Note: GAF = Global Assessment of Functioning; CGI-S = Clinical Global Impression-Severity. Continuous data are presented as mean ± standard deviation (median), and categorical data are presented as n (%). Negative values in CGI-S Score Change indicate an improvement in illness severity.)
